# Unipolar Parity of Ferroelectric-Antiferroelectric Characterized by Junction Current in Crystalline Phase Hf_1−x_Zr_x_O_2_ Diodes

**DOI:** 10.3390/nano11102685

**Published:** 2021-10-12

**Authors:** Kuo-Yu Hsiang, Chun-Yu Liao, Jer-Fu Wang, Zhao-Feng Lou, Chen-Ying Lin, Shih-Hung Chiang, Chee-Wee Liu, Tuo-Hung Hou, Min-Hung Lee

**Affiliations:** 1 Institute and Undergraduate Program of Electro-Optical Engineering, National Taiwan Normal University, Taipei 11677, Taiwan; 60648013s@ntnu.edu.tw (K.-Y.H.); 60548043s@ntnu.edu.tw (C.-Y.L.); 60977024h@ntnu.edu.tw (Z.-F.L.); 60977005h@ntnu.edu.tw (C.-Y.L.); 60877017h@ntnu.edu.tw (S.-H.C.); 2 Department of Electronics Engineering and Institute of Electronics, National Yang Ming Chiao Tung University, Hsinchu 300, Taiwan; thhou@mail.nctu.edu.tw; 3 Graduate Institute of Electronics Engineering, National Taiwan University, Taipei 10617, Taiwan; D03943022@ntu.edu.tw (J.-F.W.); chee@cc.ee.ntu.edu.tw (C.-W.L.)

**Keywords:** ferroelectric, antiferroelectric, HfZrO_2_

## Abstract

Ferroelectric (FE) Hf_1−x_Zr_x_O_2_ is a potential candidate for emerging memory in artificial intelligence (AI) and neuromorphic computation due to its non-volatility for data storage with natural bi-stable characteristics. This study experimentally characterizes and demonstrates the FE and antiferroelectric (AFE) material properties, which are modulated from doped Zr incorporated in the HfO_2_-system, with a diode-junction current for memory operations. Unipolar operations on one of the two hysteretic polarization branch loops of the mixed FE and AFE material give a low program voltage of 3 V with an ON/OFF ratio >100. This also benefits the switching endurance, which reaches >10^9^ cycles. A model based on the polarization switching and tunneling mechanisms is revealed in the (A)FE diode to explain the bipolar and unipolar sweeps. In addition, the proposed FE-AFE diode with Hf_1−x_Zr_x_O_2_ has a superior cycling endurance and lower stimulation voltage compared to perovskite FE-diodes due to its scaling capability for resistive FE memory devices.

## 1. Introduction

High-density and low-power consumption devices are in high demand for the enablement of artificial intelligence/machine learning (AI/ML) and neuromorphic computation [1]. The development of traditional memory technologies satisfies the well-known ‘memory wall’ problem [2]. Various emerging non-volatile memory (NVM) technologies have been proposed to solve this problem because of their high density, low power, fast access speed, low latency, non-volatility, etc. [3]. Such NVM technologies include resistive random-access memory (RRAM), phase-change memory (PCM), ferroelectric Field-Effect Transistors (FET), and capacitors [4,5,6,7,8,9,10]. Among them, ferroelectric Hf-based oxides have been given wide attention for applications such as negative capacitance (NC) FETs, non-volatile FeFET, 3D ferroelectric capacitors, ferroelectric diode, and ferroelectric tunnel junctions (FTJ) [11,12,13,14,15,16,17,18,19,20,21,22,23,24,25,26].

Resistive ferroelectric-based memory has attracted great interest for memory devices due to its non-volatility for data storage, non-destructive readout, and high switching speed [27,28,29,30]. The metal/ferroelectric/metal (MFM) structure of resistive ferroelectric capacitors (FeCAPs) is composed of a ferroelectric (FE) thin film sandwiched between two metal electrodes. Two non-volatile resistance states, the low-resistance state (LRS) and the high-resistance state (HRS), can be programmed by applying pulse voltages with opposite polarities to modulate the polarizations in FE films. The FeCAP currents constitute several current mechanisms, including the tunneling current, ferroelectric current, and capacitive current. The tunneling probability of the diode is dictated by the oxide thickness and the applied voltage. The displacement charge exchange of ferroelectric dipole switching and paraelectric capacitance is induced from an E-field sweep.

Furthermore, anti-ferroelectric (AFE) Hf_1−x_Zr_x_O_2_ (HZO) is reported to provide a faster switching speed for polarization and a higher fatigue resistance than FE HZO under bipolar electrical cycling [31,32,33]. In addition, lead zirconate titanate (PZT)-based AFE materials applied with a unipolar voltage were reported to show unipolar polarization-electric field (P-E) hysteresis loops [34]. Recently, an FTJ with AFE PbZrO_3_ was reported to have a high tunneling electroresistance with the potential for low-power and high-performance electronic devices [35]. The unipolar hysteresis loop for AFE-HZO with built-in bias by work function difference of electrodes was used in anti-ferroelectric random-access memory (RAM) to achieve an endurance of more than 10^9^ cycles, which is higher than ferroelectric RAM [36].

In this work, the mechanism and performance of the (A)FeCAP junction current is discussed with Hf_1−x_Zr_x_O_2_ from the FE-type to the AFE-type. The Hf:Zr ratio is adjusted to modulate the ferroelectric orthorhombic (o) phase for the type transitions. The current operational mechanism for the AFE diode with one of these two hysteretic branches is discussed, while the high ratio of the HRS/LRS and switching endurance are demonstrated. The CMOS process compatibility and scaling capability of Hf_1−x_Zr_x_O_2_ enable emerging memory applications for AI and neuromorphic computation devices.

## 2. Materials and Methods

**Figure 1:**
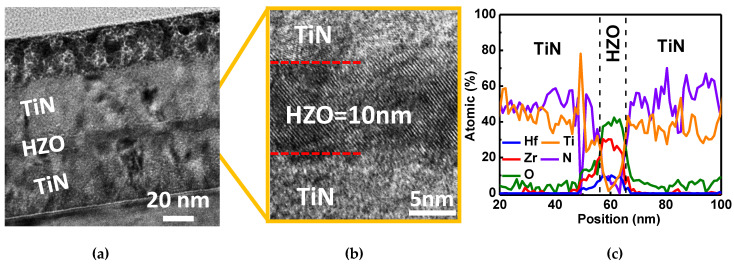
(**a**) Cross-sectional HR-TEM image of the TiN/HZO/TiN stack, (**b**) magnified of stack structure to enlarge and identify the HZO thickness 10 nm and the polycrystalline morphology after annealing. (**c**) EDS of TiN/HZO/TiN. The elements and thicknesses agree with the nominal condition of device fabrication.

**Figure 2:**
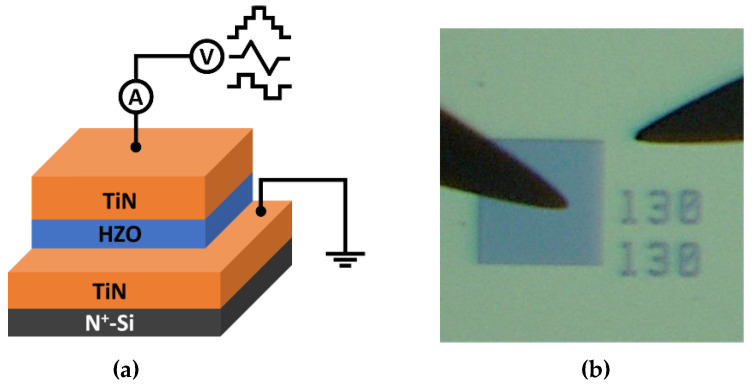
(**a**) The schematic diagram of the measurement setup, where the input voltage has DC, AC, and pulse mode. (**b**) The device pattern for measurement by optical microscope.

**Figure 3:**
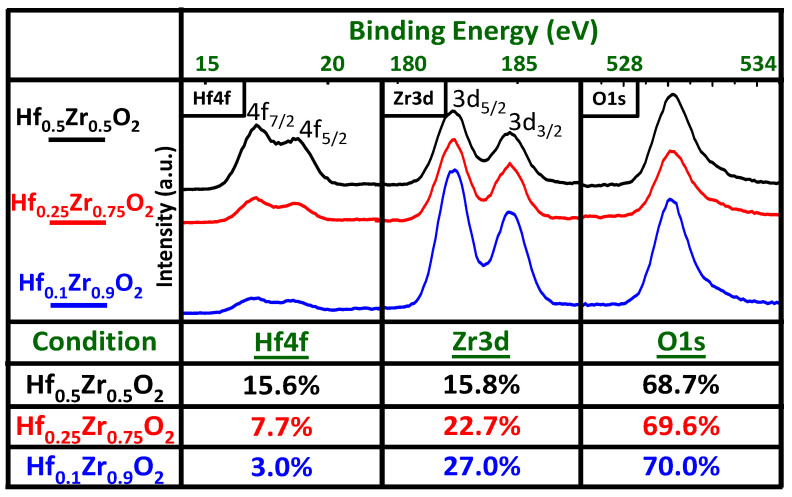
The XPS of the HZOs for Hf:Zr at 15.6:15.8 ([Zr] = 50%), 7.7:22.7 ([Zr] = 75%), and 3:27 ([Zr] = 90%). The Hf:Zr in the HZOs spectra agrees with the nominal ALD cycle ratio.

A 50 nm TiN is deposited on an N^+^-Si substrate as the bottom electrode (BE). Then, HZO films are deposited on TiN using atomic layer deposition (ALD) at 250 °C and controlled by repeatedly varying the ALD cycle ratios for HfO_2_ and ZrO_2_, whose precursors are Tetrakis(dimethylamino)hafnium (TDMA-Hf) and Tetrakis(dimethylamino)zirconium TDMA-Zr, respectively. The oxidant gas H_2_O is inserted between each cycle of HfO_2_ and ZrO_2_. A 50 nm TiN top electrode (TE) is covered on the previous HZO layer using a sputtering system. The HZO is crystallized after rapid thermal annealing (RTA) in ambient Ar at 500 °C for 1 min. The FeCAP diode shows the sandwich structure of TiN/HZO/TiN and HZO with a thickness of 10 nm and the crystallization of HZO after annealing as validated using transmission electron microscopy (TEM) (JEOL USA Inc., USA), as shown in Figure 1a,b. Figure 1c shows the energy dispersive spectroscopy (EDS) of TiN/HZO/TiN. The elements and thicknesses agree with the nominal condition of device fabrication. Figure 2a shows the schematic diagram of the measurement setup, where the input voltage has DC, AC, and pulse mode, and Figure 2b shows the device pattern for measurement by optical microscope. In this work, the HfO_2_:ZrO_2_ cycle is nominally 1:1, 1:3, and 1:9, and the atomic composition ratios are confirmed using X-ray photoelectron spectroscopy (XPS) for Hf:Zr of 15.6:15.8 ([Zr] = 50%), 7.7:22.7 ([Zr] = 75%), and 3:27 ([Zr] = 90%), respectively, as shown in Figure 3. The Hf:Zr in HZOs from the XPS agrees with the nominal cycle ratio.

**Figure 4:**
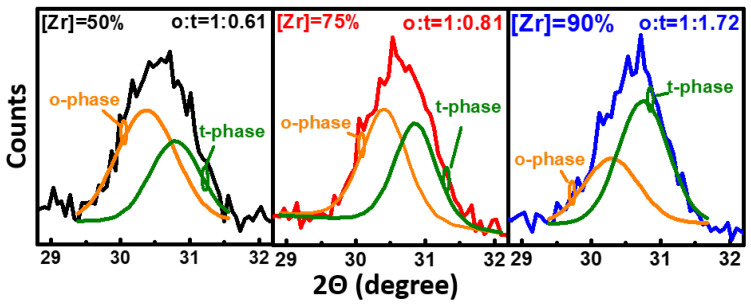
The grazing-incidence XRD (GIXRD) pattern of the HZOs for the [Zr] = 50%, 75%, and 90% diodes. The ratios of the o:t phases are extracted as 1:0.61, 1:0.81, and 1:1.72, respectively, by fitting the peak at 30–31°.

The crystalline phase of HZO after RTA is confirmed using grazing incidence X-ray diffraction (GIXRD), as presented in Figure 4. The orthorhombic (o) and tetragonal (t) phases are believed to locate at 30–31°, while the cubic (c) phase is neglected as its formation temperature is above 1000 °C, which is far higher than the RTA temperature in this study [37]. The ratio of the o:t phase is extracted as 1:0.61, 1:0.81, and 1:1.72 for [Zr] = 50%, 75%, and 90%, respectively, by fitting the peak at 30–31°. A higher t-phase and lower ferroelectric o-phase are observed with increasing amount of doped Zr in HfO_2_ in the HZO system [38]. Moreover, the crystalline grain size is revealed by the Scherrer equation, where a smaller full width at half maximum (FWHM) indicates an enhanced crystallinity [39]. The grain sizes of HZO with [Zr] = 50%, 75%, and 90% are similar at 7.6, 7.8, and 8.0 nm, respectively. 

## 3. Results

### 3.1. Junction Current Composition of FeCAPs

**Figure 5:**
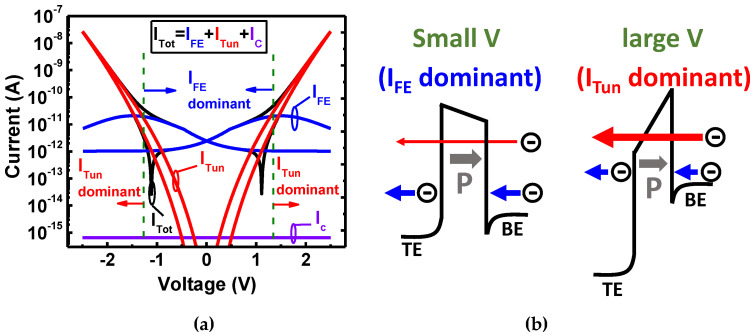
(**a**) Modelling and (**b**) schematic band diagrams for the current composition of HZO with [Zr] = 50%. The *I_tot_* is composed of the low *I_Tun_* and the high *I_Tun_* for the small and large bias, respectively.

The junction current (*I_tot_*) characteristics are composed of the mechanisms for the ferroelectric-based dipole (*I_FE_*), capacitance displacement (*I_C_*), and tunneling-based current (*I_Tun_*), which are given as

(1) $$I_{tot}\;=\;I_{FE}+I_{Tun}+I_{C}$$

(2) $$I_{FE}\;=\;A\frac{dP_{FE}}{dt}$$

(3) $$I_{C}\;=\;AC\frac{dV}{dt}$$

(4) $$I_{Tun}\;=\;A\frac{4\pi m_{eff}q}{h^{3}}\int_{E_{min}}^{E_{max}}T\left(E_{x}\right)N\left(E_{x}\right)dE_{x}\;=\;I_{DT}+I_{FN}$$

where *I_FE_* and *I_C_* are the time-dependent ferroelectric-based polarization current and the capacitance current, respectively [40]. The tunneling component is based on the Wentzel–Kramers–Brillouin (WKB) approximation, where *T(E_x_)*, *N(E_x_), m_eff_* and *h* are the transmission coefficient, the supply function, the effective mass, and the Planck constant, respectively [41]. The tunneling mechanism mainly involves the direct tunneling (*I_DT_*) and the Fowler–Nordheim tunneling (*I_FN_*). The modelling and schematic band diagrams for the current composition of HZO with [Zr] = 50% are shown in Figure 5a,b, respectively. For the small applied voltage, the electron transmission probability of direct tunneling is quite small due to the comparatively thick HZO (10 nm). The ferroelectric-based current would dominate this region, which is caused by the polarization dipole switching with the bias gradually increasing, as shown in Figure 5b. Once the large voltage is applied, the severe band bending contributes to the electron transmission probability by Fowler–Nordheim tunneling and leads to *I_Tun_* domination, as shown in Figure 5b. Note that the capacitance displacement current is ignored due to it being relatively small in comparison with the other two components [40].

**Figure 6:**
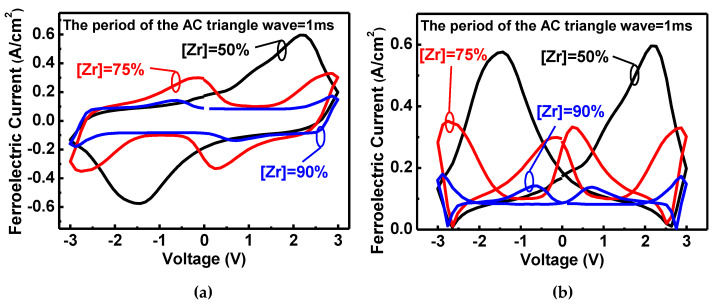
The AC-based ferroelectric polarization current of (A)FeCAPs for (**a)** raw data, and (**b**) the absolute value (amplitude only). The orthorhombic phase and the switchable dipoles of (A)FeCAPs are decreased with Zr composition enhancement that causes the *I_FE_* to be decreased under bipolar operation.

In order to characterize the ferroelectric-based current and suppress the non-ferroelectric polarization current, i.e., eliminate the tunneling current, the (A)FeCAPs are operated on fast AC triangle wave with a period of 1ms. Figure 6 shows the ferroelectric polarization current. The orthorhombic phase and the switchable dipoles of AFeCAPs are decreased, which leads to a decrease in *I_FE_* with high Zr composition. Note that the cross points of Figure 6b indicate the equal of the positive and negative currents in Figure 6a, which is the balance bias toward the dipoles gradually switching. In this work, the base voltage for subsequent operation of memory characteristics is according to this concept. For the LRS, the polarization dipole switching contributes to the high *I_FE_* response. In contrast, HRS exhibits the low ferroelectric current with dipole non-switching.

### 3.2. Dipole Switch Characteristics of (A)FeCAPs by Bipolar Bias

**Figure 7:**
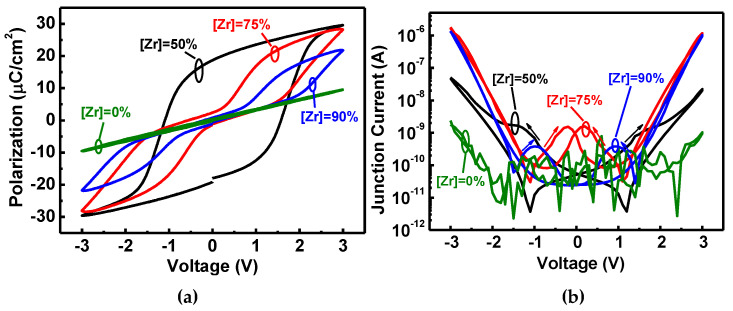
The HZOs with the [Zr] = 0%, 50%, 75%, and 90% diodes for the double bipolar sweep (−3 V to 3 V and back to −3 V) for the (**a**) P-V and (**b**) I-V characteristics.

Figure 7a,b shows the hysteresis loop (P-V) and junction current–voltage (I-V) characteristics for HZO (A)FeCAPs, and pure HfO_2_, respectively, under bipolar bias operations for the [Zr] = 0%, 50%, 75%, and 90% diodes with a double sweep (−3 to 3 V and back to −3 V). The P-V and I-V of the pure HfO_2_ (Zr = 0%) with non-ferroelectric show a typical paraelectric behavior. The FeCAP ([Zr] = 50%) has the highest remnant polarization (P_r_), compared with the AFeCAPs ([Zr] = 75% and 90%), in Figure 7a. The P_r_ and the saturation polarization (P_s_) with [Zr] = 75% are slightly higher than [Zr] = 90% due to the partial o-phase. The P-V of AFeCAPs ([Zr] = 75% and 90%) has double hysteresis loop branch characteristics, which have multiple peaks in the I-V curves of Figure 7b due to multiple stages of gradual polarization.

### 3.3. Dipole Switch Characteristics of (A)FeCAPs with Unipolar Bias

**Figure 8:**
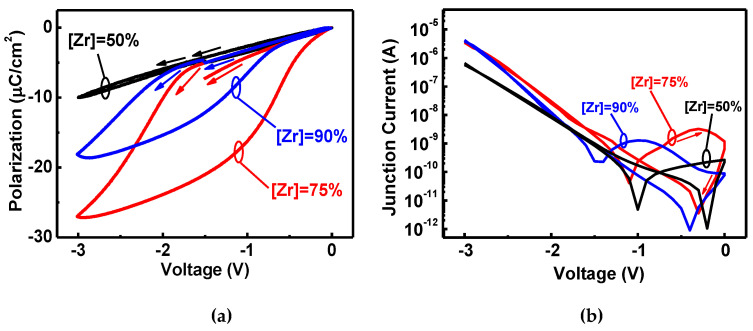
The HZOs with the [Zr] = 50%, 75%, and 90% diodes for the unipolar sweep (−3 V to 0 and back to −3 V) for the (**a**) P-V and (**b**) I-V characteristics. The [Zr] = 75% and [Zr] = 90% for the AFeCAPs exhibit the “typical” ferroelectric behavior with unipolar operation. There is paraelectric behavior for [Zr] = 50%. The much higher polarization and ferroelectric-based current are obtained for AFE diodes with unipolar operation.

Figure 8a,b show the P-V and junction current–voltage (I-V) characteristics of the HZO diodes under unipolar bias operation for [Zr] = 50%, 75%, and 90% with a double sweep (−3 to 0 V and back to −3 V). The [Zr] = 50% diode exhibits nearly paraelectric behavior with no polarization switching in the unipolar operational range (0 to −3 V) in Figure 8a. In contrast, the other two diodes at [Zr] = 75% and [Zr] = 90% under unipolar operations preserve one of the branches of its double hysteresis loop. Similarly, the I-V curves for [Zr] = 75% and [Zr] = 90% have FE characteristics under unipolar operations in Figure 7b.

### 3.4. Memory Characteristics of (A)FeCAPs by Program/Erase Pulse Stimulation

**Figure 9:**
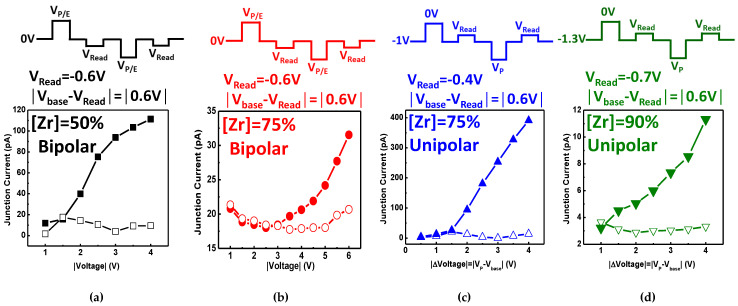
The ON and OFF currents under bipolar operation for the (**a**) FE diode [Zr] = 50% and (**b**) FE-AFE diode [Zr] = 75%, and unipolar operation for the (**c**) FE-AFE diode [Zr] = 75% and (**d**) AFE diode [Zr] = 90% with various V_P/E_. The results agree with the current sweep (**c**,**d**), and indicate that the FE-AFE mixture of [Zr] = 75% is conducive as a high current ratio to enhance memory state discrimination.

To apply the (A)FE junction current for memory applications, read-out from sensing the current sampling measurements with the program/erase (P/E) pulse stimulation is performed. Figure 9a,b shows the ON and OFF currents under bipolar operations for the [Zr] = 50% and [Zr] = 75% diodes. Figure 9c,d presents unipolar operations for the [Zr] = 75% and [Zr] = 90% diodes with various V_P/E_. The pulse sequences for the read and write (program/erase) operations are illustrated. For bipolar operations of a simple FE diode, the V_P/E_ is applied to switch the polarization and set the LRS (V_P/E_ > 0) or HRS (V_P/E_ < 0) states when reading the negative interval. A small read voltage of −0.6V is chosen to avoid distorting the stored state, which is smaller than the coercive voltage. In Figure 9a,b, the [Zr] = 50% diode exhibits a higher ON/OFF current ratio due to the stronger polarization (Figure 6b and Figure 7b) compared with [Zr] = 75%. However, [Zr] = 75% and 90% diodes are like volatile memory that is not suitable for bipolar operations.

For unipolar operations, a constant base voltage (V_base_) is needed to retain the information of the AFeCAPs, and the write voltage V_P_ is applied to switch the polarization to the LRS (V_P_ < 0) or HRS (V_P_ = 0) state. Note that, the base voltage corresponds to the cross points in Figure 8b, and the base voltage of [Zr] = 75% and 90% are −1 V and −1.3 V, respectively. The read voltage is set to 0.6 V (|V_base_-V_read_|) to avoid distorting the stored state. A larger current ratio for the [Zr] = 75% diode between the HRS and LRS states under unipolar operations is observed in Figure 9c,d compared with the [Zr] = 90%. Note that the constant |V_base_-V_read_| for all measurements (both bipolar and unipolar operation) is applied to control the same read condition. Since the coercive voltage of unipolar-based [Zr] = 75% is smaller than that of bipolar-based [Zr] = 50%, the ferroelectric current ratio of the former is larger than the latter. The results agree with the current sweep (Figure 6b and Figure 8a,b), which indicates that the FE-AFE mixture for the [Zr] = 75% is conducive for high current ratios to enhance the memory state discrimination. Furthermore, unipolar operation also benefits a reduced operational voltage range, i.e., ΔV_P/E_ = 6 V (bipolar) to 3 V (unipolar), which indicates an effective improvement on power consumption.

### 3.5. Memory Reliability of (A)FeCAPs

**Figure 10:**
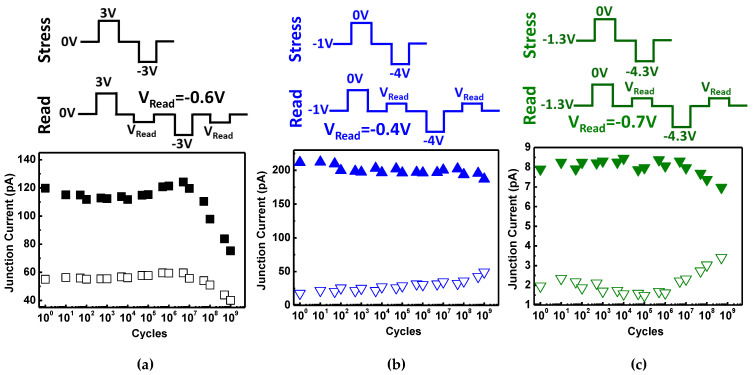
The endurance characteristics of the diodes for (**a**) [Zr] = 50% under bipolar operations, and (**b**) [Zr] = 75% and (**c**) [Zr] = 90% under unipolar operations. Unipolar operations for the AFE diodes show superior endurance, especially the [Zr] = 75%, which exhibits no obvious degradation up to 10^9^ cycles. The FE diode with bipolar switching degraded significantly after 10^7^ cycles of AC stress.

Another advantage of unipolar operations is an improved switching endurance. Figure 10a–c shows the switching endurance characteristics for the [Zr] = 50% diode under bipolar operations, and the [Zr] = 75% and [Zr] = 90% diodes under unipolar operations, respectively, with a cycle number that approaches 10^9^. The measurement sequences for each device illustrate that the V_P/E_ is higher than the V_c_ to switch the dipoles and set the HRS or LRS. A small V_read_ of 0.6 V is applied to avoid distorting the stored state. Note that the ferroelectric current in the sampling of [Zr] = 90% is lower than [Zr] = 75% due to polarization magnitude in Figure 8a. The simple FE diode ([Zr] = 50%) under bipolar switching degraded significantly after 10^7^ cycles of AC switching stress. Unipolar operation of the AFeCAPs shows a superior endurance, especially for [Zr] = 75%, which exhibits no obvious degradation up to 10^9^ cycles. The fatigue mechanisms of ferroelectric layer are attributed to polarization degradation with domain pinning and/or nucleation inhibition for switch cycling [42]. The oxygen vacancies at the interface between the HZO and the TiN electrode benefit the t-phase stability, i.e., suppress the m-phase formation [33,43]. The excessive oxygen vacancies accumulation would lead to a breakdown via strong interactions between the individual vacancies [44]. The unipolar operates in a single parity to improve the effect of charged defects or injected charges as compared with the bipolar operation. Therefore, the AFeCAPs ([Zr] = 75% and 90%) with the unipolar operation exhibits not only reducing the operating voltage but also endurance improvement. Note that higher base voltage of [Zr] = 90% makes a significant fatigue effect as compared with [Zr] = 75%. Compared with the performance of prior arts, unipolar operations and low V_P/E_ are advantageous for the proposed FE-AFE ([Zr] = 75%) diode and result in a high switching endurance of >10^9^ cycles. Table 1 shows the benchmark comparison of the characteristics of other resistive ferroelectric memory devices, perovskite FE-diodes from Refs. [24,25,26]. Ref. [25,26] had reported >10^5^ and ~10^4^ times of the ON/OFF ratio by a depletion/accumulation charge in IGZO and 3D-stackable with self-selective, respectively. Therefore, the ferroelectric HfO_2_-based material has potential toward a high ON/OFF ratio.

## 4. Conclusions

The FE and AFE diodes under bipolar and unipolar operations are characterized by their current mechanisms. The Hf:Zr ratio is adjusted to modulate the ferroelectric o-phase for the FE-type to AFE-type transition. The [Zr] = 75% diode for the HZO with a mixed o-phase and t-phase provides unipolar operational capabilities within one of the two hysteretic polarization branches and reduces the program voltage. This provides high endurance >10^9^ cycles and has a high ON/OFF ratio > 100x under low-voltage operation (0 V to −3 V). The results show that AFE materials are promising for resistive switching memory devices for AI/ML applications.

## Figures and Tables

**Table 1:** Benchmark of the comparison with prior arts for resistive ferroelectric diodes, perovskite FE-diodes for Refs. [24,25,26]. The unipolar operation and low V_P/E_ are advantages of the proposed FE-AFE diode and result in high switch endurance of >10^9^ cycles.

Reference	This Work	[24]	[25]	[26]
Material	FE-AFE HZO	FE-BFO	FE-HZO/IGZO	FE-HZO
Thickness (nm)	10	170	7/5	10
V_P_/V_E_	–3 V/0 V	+8 V/–8 V	+3.3 V/–3.2 V	+9.5 V/+5.9 V
│V_base_-V_read_│	0.6 V	4 V	2 V	2 V
I_ON_/I_OFF_	>100	~5	~3 × 10^5^	~10,000
Endurance (cycles)	>10^9^	NA	~10^9^	>10^9^

## Data Availability

The data are not publicly available due to privacy. The data present in this research are available on request from the corresponding author.

## References

[1] Wu T.-Y., Huang H.-H., Chu Y.-H., Chang C.-C., Wu M.-H., Hsu C.-H., Wu C.-T., Wu M.-C., Wu W.-W., Chang T.-S. Sub-nA low-current HZO ferroelectric tunnel junction for high-performance and accurate deep learning acceleration. Proceedings of the 2019 IEEE International Electron Devices Meeting (IEDM).

[2] Jacob P., Zia A., Erdogan O., Belemjian P.M., Kim J.-W., Chu M., Kraft R.P., McDonald J.F., Bernstein K. (2009). Mitigating memory wall effects in high-clock-rate and multicore CMOS 3-D processor memory stacks. Proc. IEEE.

[3] Sun G., Zhao J., Poremba M., Xu C., Xie Y. (2018). Memory that never forgets: Emerging nonvolatile memory and the implication for architecture design. Natl. Sci. Rev..

[4] Woo J., Moon K., Song J., Lee S., Kwak M., Park J., Hwang H. (2016). Improved synaptic behavior under identical pulses using AlO_x_/HfO_2_ bilayer RRAM array for neuromorphic systems. IEEE Electron. Device Lett..

[5] Yu S., Chen P.-Y., Cao Y., Xia L., Wang Y., Wu H. Scaling-up resistive synaptic arrays for neuro-inspired architecture: Challenges and prospect. Proceedings of the 2015 IEEE International Electron Devices Meeting (IEDM).

[6] Burr G.W., Shelby R.M., Sidler S., Di Nolfo C., Jang J., Boybat I., Shenoy R.S., Narayanan P., Virwani K., Giacometti E.U. (2015). Experimental demonstration and tolerancing of a large-scale neural network (165,000 synapses) using phase-change memory as the synaptic weight element. IEEE Trans. Electron. Devices.

[7] Jerry M., Chen P.-Y., Zhang J., Sharma P., Ni K., Yu S., Datta S. Ferroelectric FET analog synapse for acceleration of deep neural network training. Proceedings of the 2017 IEEE International Electron Devices Meeting (IEDM).

[8] Seo M., Kang M.-H., Jeon S.-B., Bae H., Hur J., Jang B.C., Yun S., Cho S., Kim W.-K., Kim M.-S. (2018). First demonstration of a logic-process compatible junctionless ferroelectric FinFET synapse for neuromorphic applications. IEEE Electron. Device Lett..

[9] Oh S., Kim T., Kwak M., Song J., Woo J., Jeon S., Yoo I.K., Hwang H. (2017). HfZrO x-based ferroelectric synapse device with 32 levels of conductance states for neuromorphic applications. IEEE Electron. Device Lett..

[10] Hu V.P.-H., Lin H.-H., Zheng Z.-A., Lin Z.-T., Lu Y.-C., Ho L.-Y., Lee Y.-W., Su C.-W., Su C.-J. Split-gate FeFET (SG-FeFET) with dynamic memory window modulation for non-volatile memory and neuromorphic applications. Proceedings of the 2019 Symposium on VLSI Technology.

[11] Böscke T., Müller J., Bräuhaus D., Schröder U., Böttger U. Ferroelectricity in hafnium oxide: CMOS compatible ferroelectric field effect transistors. Proceedings of the 2011 International Electron Devices Meeting.

[12] Polakowski P., Riedel S., Weinreich W., Rudolf M., Sundqvist J., Seidel K., Muller J. Ferroelectric deep trench capacitors based on Al: HfO_2_ for 3D nonvolatile memory applications. Proceedings of the 2014 IEEE 6th International Memory Workshop (IMW).

[13] Cheng C.-H., Chin A. (2013). Low-leakage-current DRAM-like memory using a one-transistor ferroelectric MOSFET with a Hf-based gate dielectric. IEEE Electron. Device Lett..

[14] Cheng C.H., Chin A. (2014). Low-Voltage Steep Turn-On pMOSFET Using Ferroelectric High-k Gate Dielectric. IEEE Electron. Device Lett..

[15] Park M.H., Kim H.J., Kim Y.J., Moon T., Do Kim K., Hwang C.S. (2015). Toward a multifunctional monolithic device based on pyroelectricity and the electrocaloric effect of thin antiferroelectric Hf_x_Zr_1−x_O_2_ films. Nano Energy.

[16] Chiu Y.-C., Cheng C.-H., Chang C.-Y., Lee M.-H., Hsu H.-H., Yen S.-S. Low power 1T DRAM/NVM versatile memory featuring steep sub-60-mV/decade operation, fast 20-ns speed, and robust 85 C-extrapolated 10^16^ endurance. Proceedings of the 2015 Symposium on VLSI Technology (VLSI Technology).

[17] Fujii S., Kamimuta Y., Ino T., Nakasaki Y., Takaishi R., Saitoh M. First demonstration and performance improvement of ferroelectric HfO_2_-based resistive switch with low operation current and intrinsic diode property. Proceedings of the 2016 IEEE Symposium on VLSI Technology.

[18] Mulaosmanovic H., Ocker J., Müller S., Noack M., Müller J., Polakowski P., Mikolajick T., Slesazeck S. Novel ferroelectric FET based synapse for neuromorphic systems. Proceedings of the 2017 Symposium on VLSI Technology.

[19] Eskandari R., Zhang X., Malkinski L.M. (2017). Polarization-dependent photovoltaic effect in ferroelectric-semiconductor system. Appl. Phys. Lett..

[20] Dragoman M., Aldrigo M., Modreanu M., Dragoman D. (2017). Extraordinary tunability of high-frequency devices using Hf_0.3_Zr_0.7_O_2_ ferroelectric at very low applied voltages. Appl. Phys. Lett..

[21] Van Houdt J. Memory technology for the terabit era: From 2D to 3D. Proceedings of the 2017 Symposium on VLSI Technology.

[22] Smith S., Kitahara A., Rodriguez M., Henry M., Brumbach M., Ihlefeld J. (2017). Pyroelectric response in crystalline hafnium zirconium oxide (Hf_1−x_ Zr_x_ O_2_) thin films. Appl. Phys. Lett..

[23] Huang F., Wang Y., Liang X., Qin J., Zhang Y., Yuan X., Wang Z., Peng B., Deng L., Liu Q. (2017). HfO_2_-based highly stable radiation-immune ferroelectric memory. IEEE Electron. Device Lett..

[24] Chen Z., He L., Zhang F., Jiang J., Meng J., Zhao B., Jiang A. (2013). The conduction mechanism of large on/off ferroelectric diode currents in epitaxial (111) BiFeO_3_ thin film. J. Appl. Phys..

[25] Bae H., Moon T., Nam S.G., Lee K.-H., Kim S., Hong S., Choe D.-H., Jo S., Lee Y., Heo J. Ferroelectric Diodes with Sub-ns and Sub-fJ Switching and Its Programmable Network for Logic-in-Memory Applications. Proceedings of the 2021 Symposium on VLSI Technology.

[26] Luo Q., Cheng Y., Yang J., Cao R., Ma H., Yang Y., Huang R., Wei W., Zheng Y., Gong T. (2020). A highly CMOS compatible hafnia-based ferroelectric diode. Nat. Commun..

[27] Kobayashi M., Tagawa Y., Mo F., Saraya T., Hiramoto T. (2018). Ferroelectric HfO_2_ tunnel junction memory with high TER and multi-level operation featuring metal replacement process. IEEE J. Electron. Devices Soc..

[28] Garcia V., Fusil S., Bouzehouane K., Enouz-Vedrenne S., Mathur N.D., Barthelemy A., Bibes M. (2009). Giant tunnel electroresistance for non-destructive readout of ferroelectric states. Nature.

[29] Boyn S., Chanthbouala A., Girod S., Carrétéro C., Barthélémy A., Bibes M., Grollier J., Fusil S., Garcia V. (2018). Real-time switching dynamics of ferroelectric tunnel junctions under single-shot voltage pulses. Appl. Phys. Lett..

[30] Hsiang K.-Y., Liao C.-Y., Chen K.-T., Lin Y.-Y., Chueh C.-Y., Chang C., Tseng Y.-J., Yang Y.-J., Chang S., Liao M.-H. (2020). Ferroelectric HfZrO _2_ with electrode engineering and stimulation schemes as symmetric analog synaptic weight element for deep neural network training. IEEE Trans. Electron. Devices.

[31] Lyu X., Si M., Sun X., Capano M., Wang H., Ye P. Ferroelectric and anti-ferroelectric hafnium zirconium oxide: Scaling limit, switching speed and record high polarization density. Proceedings of the 2019 Symposium on VLSI Technology.

[32] Lou X. (2009). Why do antiferroelectrics show higher fatigue resistance than ferroelectrics under bipolar electrical cycling?. Appl. Phys. Lett..

[33] Hsiang K.-Y., Liao C.-Y., Liu J.-H., Wang J.-F., Chiang S.-H., Chang S.-H., Hsieh F.-C., Liang H., Lin C.-Y., Lou Z.-F. (2021). Bilayer-based Antiferroelectric HfZrO_2_ Tunneling Junction with High Tunneling Electroresistance and Multilevel Nonvolatile Memory. IEEE Electron. Device Lett..

[34] Gao M., Tang X., Leung C.M., Dai S., Li J., Viehland D.D. (2019). Phase transition and energy storage behavior of antiferroelectric PLZT thin films epitaxially deposited on SRO buffered STO single crystal substrates. J. Am. Ceram. Soc..

[35] Apachitei G., Peters J.J., Sanchez A.M., Kim D.J., Alexe M. (2017). Antiferroelectric tunnel junctions. Adv. Electron. Mater..

[36] Pesic M., Knebel S., Hoffmann M., Richter C., Mikolajick T., Schroeder U. How to make DRAM non-volatile? Anti-ferroelectrics: A new paradigm for universal memories. Proceedings of the 2016 IEEE International Electron Devices Meeting (IEDM).

[37] Kumar N., George B.P.A., Abrahamse H., Parashar V., Ray S.S., Ngila J.C. (2017). A novel approach to low-temperature synthesis of cubic HfO _2_ nanostructures and their cytotoxicity. Sci. Rep..

[38] Lee M.-H., Wei Y.-T., Chu K.-Y., Huang J.-J., Chen C.-W., Cheng C.-C., Chen M.-J., Lee H.-Y., Chen Y.-S., Lee L.-H. (2015). Steep slope and near non-hysteresis of FETs with antiferroelectric-like HfZrO for low-power electronics. IEEE Electron. Device Lett..

[39] Monshi A., Foroughi M.R., Monshi M.R. (2012). Modified Scherrer equation to estimate more accurately nano-crystallite size using XRD. World J. Nano Sci. Eng..

[40] Huang H.-H., Wu T.-Y., Chu Y.-H., Wu M.-H., Hsu C.-H., Lee H.-Y., Sheu S.-S., Lo W.-C., Hou T.-H. A comprehensive modeling framework for ferroelectric tunnel junctions. Proceedings of the 2019 IEEE International Electron Devices Meeting (IEDM).

[41] Yılmaz K., Farokhnejad A., Criado F., Iñíguez B., Lime F., Kloes A. Direct source-to-drain tunneling current in ultra-short channel DG MOSFETs by wavelet transform. Proceedings of the 2020 IEEE Latin America Electron Devices Conference (LAEDC).

[42] Song T., Estandía S., Tan H., Dix N., Gàzquez J., Fina I., Sánchez F. (2021). Positive Effect of Parasitic Monoclinic Phase of Hf_0.5_Zr_0.5_O_2_ on Ferroelectric Endurance. Adv. Electron. Mater..

[43] Kim B.S., Hyun S.D., Moon T., Do Kim K., Lee Y.H., Park H.W., Lee Y.B., Roh J., Kim B.Y., Kim H.H. (2020). A Comparative Study on the Ferroelectric Performances in Atomic Layer Deposited Hf_0.5_ Zr_0.5_ O_2_ Thin Films Using Tetrakis (ethylmethylamino) and Tetrakis (dimethylamino) Precursors. Nanoscale Res. Lett..

[44] Hoffmann M., Schroeder U., Schenk T., Shimizu T., Funakubo H., Sakata O., Pohl D., Drescher M., Adelmann C., Materlik R. (2015). Stabilizing the ferroelectric phase in doped hafnium oxide. J. Appl. Phys..

